# Influence of Treatment Temperature on Microstructure and Properties of YSZ–NiO Anode Materials

**DOI:** 10.1186/s11671-016-1306-z

**Published:** 2016-02-16

**Authors:** Viktoriya Podhurska, Bogdan Vasyliv, Orest Ostash, Yegor Brodnikovskyi, Oleksandr Vasylyev

**Affiliations:** H. V. Karpenko Physico-Mechanical Institute of the National Academy of Sciences of Ukraine, 5 Naukova str, Lviv, 79060 Ukraine; I. M. Frantsevich Institute for Problems of Materials Science of the National Academy of Sciences of Ukraine, 3 Krzhizhanovsky str, Kyiv, 03680 Ukraine

**Keywords:** SOFC anode material, Hydrogenous environment, High temperature, Redox treatment, Microstructure, Strength, Electrical conductivity

## Abstract

The cyclic treatment technique (redox cycling) comprising stages of material exposition in reducing and oxidizing high-temperature environments and intermediate degassing between these stages has been developed to improve the structural integrity of YSZ–NiO ceramic anode substrates for solid oxide fuel cells. A series of specimens were singly reduced in a hydrogenous environment (the Ar–5 vol% Н_2_ mixture or hydrogen of 99.99 vol% H_2_ purity) under the pressure of 0.15 MPa or subjected to redox cycling at 600 or 800 °C. The influence of redox cycling at the treatment temperatures of 600 and 800 °C on the structure, strength and electrical conductivity of the material has been analysed. Using the treatment temperature 600 °C, a structure providing improved physical and mechanical properties of the material was formed. However, at the treatment temperature 800 °C, an anode structure with an array of microcracks was formed that significantly reduced the strength and electrical conductivity of the material.

## Introduction

A number of publications predict a dual influence of operating temperature on resulting physical and mechanical properties of the Ni-containing anode material for solid oxide fuel cells (SOFCs) after cyclic reduction–oxidation (redox) treatment [1–3]. It is well known that the electrical conductivity of metallic Ni (of about 1.4 · 10^7^ S/m) is much higher than that of YSZ–Ni cermet. According to our data, after exposition of the ceramics sintered of NiO powder, for 4 h or more in pure hydrogen at 600 °C, complete reduction can be achieved [4]. The resulting electrical conductivity of the material is about (1–5) · 10^6^ S/m. However, exposition of NiO ceramics at this regime in the Ar–5 vol% Н_2_ mixture that can be used for gradual reduction of SOFC anodes causes partial reduction of the NiO particles forming thin edgings of metallic Ni (of thickness of 0.1–0.3 μm) around them. Thus, the electrical conductivity of the material treated is in the range of (1–5) · 10^5^ S/m depending on average particle size, porosity and resulting contacts between Ni edgings. During redox treatment of NiO ceramics, structural transformation of boundaries of contacting nickel phase particles occurs, causing an increase in strength.

In our previous works, it was revealed for ScCeSZ–NiO anode ceramics that at selected redox treatment regimes when the material is heated in vacuum and intermediate degassing between reduction and oxidation stages is performed, substantial improvements in strength (up to 112 %) and electrical conductivity can be reached at the treatment temperature of 600 °C [5, 6].

The aims of this work are to study the physical and mechanical behaviour of the SOFC anode material during the cyclic redox treatment depending on the treatment temperature and also to find out the microstructural changes causing resulting properties of the material.

## Review

### Experimental

**Fig. 1:**
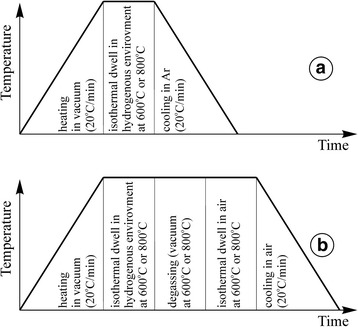
The treatment schemes applied for YSZ–NiO ceramics. **a** Single reduction in a hydrogenous environment. **b** A cycle of redox treatment

Anode ceramics of the YSZ–NiO system sintered at Forschungszentrum Jülich (Germany) of zirconium oxide powder stabilized with 8 mol% Y_2_O_3_, with the addition of 50 wt% NiO, has been investigated. A series of specimens of the size of 1 × 5 × 25 mm were singly reduced at 600 or 800 °C in a hydrogenous environment (the Ar–5 vol% Н_2_ mixture or hydrogen of 99.99 vol% H_2_ purity) under the pressure of 0.15 MPa (Fig. 1a) or subjected to redox cycling (see Table 1). The redox treatment of ceramics was performed for five cycles according to the scheme [4]: heating in vacuum from 20 to 600 °C, reduction in a hydrogenous environment at 600 or 800 °C under the pressure of 0.15 MPa, degassing, oxidation in air at 600 °C and cooling down to 20 °С in air (Fig. 1b). Reduction/oxidation stage duration was chosen, taking into account the literature data on complete or partial reduction of the material [7]. After the redox cycling, reduction of materials in a hydrogenous environment for 4 h at 600 °C or for 1 h at 800 °C under the pressure of 0.15 MPa with cooling in argon was performed (see the scheme in Fig. 1a). The heating/cooling rate was 20 °C/min.

**Table 1:** The treatment regimes for the materials tested

No. of version	Environment	Treatment temperature (°C)	Reduction/oxidation stage duration (h)	Variant of treatment
1	Ar–H_2_ mixture	600	4	R
2	H_2_	600	4	R
3	Ar–H_2_ mixture/air	600	4	RO
4	H_2_/air	600	4	RO
5	Ar–H_2_ mixture	800	1	R
6	Ar–H_2_ mixture/air	800	1	RO

*R* single reduction, *RO* redox cycling

Ultimate fracture stresses of materials in the initial state (*σ*_f0_) and after the corresponding treatment (*σ*_f_) were determined during the three-point bending test of the specimens in air at 20 °C. Based on these data, the relative strength (*σ*_f_ / *σ*_f0_) of the material treated was evaluated.

The specific electrical conductivity of the material (*σ*) was determined in air at 20 °C using the Van der Pauw method. SEM microstructures and microfractographs of the specimens were investigated using the electron microscope Carl Zeiss EVO-40XVP.

The X-ray analysis was carried out using an X-ray diffractometer (Cu Кα) with Bragg–Brentano-type geometry. The average size of coherent dispersion areas of nickel phase (*D*) was calculated using the Win CSD program [8].

The spacing between the planes in the atomic lattice (*d*) of zirconia phase (line 220) was estimated using the Rietveld method [8], and the residual stresses (*σ*_r_) were evaluated using the equation

1 $$ {\sigma}_{\mathrm{r}}=-\frac{E}{\nu}\cdot \frac{d-{d}_0}{d_0} $$

where *E* is Young’s modulus and *ν* is Poisson’s ratio; the values of these parameters were selected according to [9]; *d*_0_ is the spacing between the planes in the atomic lattice of zirconia phase (line 220) for the as-received material.

Thermodynamics of reactions of nickel phase reduction and oxidation was analysed by calculating the changes in Gibbs’ free energy (Δ*G*) using standard data [10].

### Results and Discussion

#### Thermodynamics of the Reduction and Oxidation Stages

It is known [11] that in the temperature range 630–680 °C, a transition from diffusion to kinetic mechanism of oxidation occurs. Additionally, by analysing the thermodynamics of reduction and oxidation stages, it was revealed that in the temperature range 600–800 °C, the change in Gibbs’ free energy (Δ*G*) is more negative and the equilibrium constant (*К*) is considerably higher for the reaction of nickel oxidation compared to its reduction (see Table 2). According to this, we concluded that during redox cycling at higher temperature, the Ni oxidation stage becomes more faster than the NiO reduction one. Thus, the probability of retaining unreduced particles is high, if the reduction period was too short. In such a case, the resulting structure does not meet the requirements on uniformity and, finally, efficiency of an anode substrate.

**Table 2:** The data of thermodynamics of reduction and oxidation stages for the material tested

Reaction	Gibb’s free energy (Δ*G*, kJ/mol) at 600 °C	Equilibrium constant (*К*) at 600 °C	Gibb’s free energy (Δ*G*, kJ/mol) at 800 °C	Equilibrium constant (*К*) at 800 °C
Reduction	−41	2.7 · 10^2^	−43	1.3 · 10^2^
Oxidation	−159	3 · 10^9^	−145	1 · 10^7^

#### The Treatment Temperature of 600 °C

**Fig. 2:**
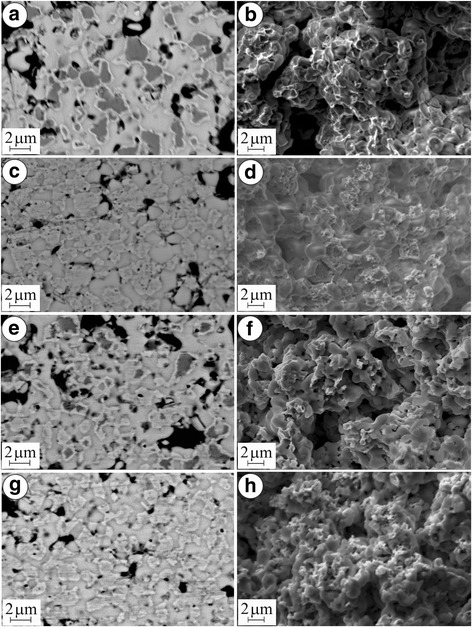
SEM microstructures (**a**, **c**, **e**, **g**) and microfractographs (**b**, **d**, **f**, **h**) for the material in versions 1 (**a**, **b**), 2 (**c**, **d**), 3 (**e**, **f**) and 4 (**g**, **h**) (see Table 1)

Exposition of YSZ–NiO ceramics at the temperature 600 °C for 4 h in the Ar–5 vol% H_2_ mixture that can be used for gradual reduction of SOFC anodes (version 1 in Table 1) causes partial reduction of the NiO particles by a diffusion mechanism. Thin Ni edgings (of thickness of 0.1–0.3 μm) are formed around NiO particles (Fig. 2а).

**Fig. 3:**
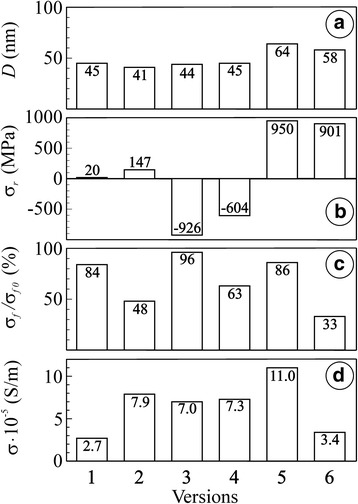
Average size of coherent dispersion area (*D*) (**a**), residual stresses (*σ*_r_), in zirconia phase (**b**), relative strength (*σ*_f_ / *σ*_f0_) (**c**) and specific electrical conductivity (*σ*) (**d**) for the material in versions 1–6 (see Table 1). The *numbers on the bars* indicate the values of corresponding parameters

The substructure of these edgings was evaluated using X-ray data on average size of coherent dispersion areas of nickel phase (*D*) (Fig. 3(а)). This parameter was measured as 45 nm.

Reduction in strength to 84 % of the value for the as-received YSZ–NiO ceramics (Fig. 3(c)) is possibly caused by partial structural transformation of nickel phase followed by a little volume decrease. It is displayed in the mixed fracture micromechanism (Fig. 2b). Although, no noticeable change of zirconia skeleton was found for this variant of the treatment. As compared to the as-received material, residual stresses did not change as well (Fig. 3(b)). The electrical conductivity of the material of 2.7 · 10^5^ S/m is provided by thin films of Ni around NiO particles united into the network (Fig. 3(d)).

More intensive reduction of YSZ–NiO ceramics by the same diffusion mechanism occurs in pure hydrogen (version 2 in Table 1). During 4 h, the structure of completely reduced Ni particles is formed (Fig. 2c). The substructure parameter (average size *D*) of these particles was measured as 41 nm that is less than for version 1 (Fig. 3(а)). Simultaneously, the volume decrease of initial NiO particles of 41.6 % occurs [12]. Nanopores on Ni particles formed due to their shrinkage as well as the pores between the particles prevent the rise of residual tensile stresses (Fig. 3(b)). Nickel phase transformation followed by volume change and formation of pores causes the loss of a significant percentage of particle bonds and violate material integrity which is displayed in the predominantly intergranular fracture micromechanism (Fig. 2d). Reduction in strength to 48 % of the value for the as-received YSZ–NiO ceramics is recognized (Fig. 3(c)). Thanks to complete reduction of nickel phase, the high electrical conductivity (Fig. 3(d)) of the material is achieved compared to the similar functional materials [13].

According to our data [4], there exists a substantial difference in the mechanical behaviour of NiO ceramics after redox treatment as compared to that of the singly reduced material, at the treatment temperature of 600 °C. During the treatment, structural transformation of boundaries of contacting nickel phase particles occurs, causing an increase in strength.

The cleavage fracture micromechanism was noted in the specimens tested. This micromechanism corresponds to the higher cohesive strength of nickel phase particles as compared to the ultimate cleavage stress of the particles themselves.

Such treatment technique has been used in this work for improvement of strength and electrical conductivity of YSZ–NiO ceramics. Upon redox cycling, exposition of the material at the temperature 600 °C for 4 h in air resulted in complete oxidation of preliminarily reduced Ni edgings on NiO particles (version 3 in Table 1, Fig. 2e) as well as of preliminarily reduced Ni particles (version 4, Fig. 2g) by diffusion mechanism. After five cycles of the redox treatment at 600 °C with final reduction stage (version 3), most of NiO particles were reduced completely, forming a continuous network of electrically conducting material in the zirconia skeleton (Fig. 2e) which resulted in the value of specific electrical conductivity of the material of 7 · 10^5^ S/m (Fig. 3(d)). Fragmentation of coarse grains of nickel phase resulted in more fine structure of the material treated. The substructure parameter (*D*) of reduced particles for version 3 was measured as 44 nm which is similar to that for version 1 (Fig. 3(а)). Thus, no distinct change of substructure of nickel particles was found.

The X-ray data displayed a reduction of 2*Θ* angle for cyclically treated materials. In accordance with Wolf–Bragg’s law

2 $$ 2d \sin \varTheta =n\lambda $$

where *n* is the positive integer and *λ* is the wavelength of incident wave; the spacing between the planes in the atomic lattice (*d*) of zirconia phase increases resulting (see Eq. (1)) in considerable relaxation of residual stresses (*σ*_r_) in the material of versions 3 and 4 as compared to the as-received material (Fig. 3(b)). This affects positively the mechanical behaviour of the material, especially of version 3 (Fig. 3(c)).

**Fig. 4:**
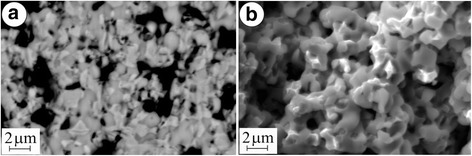
SEM microstructure (**a**) and microfractograph (**b**) for the material in version 5 (see Table 1)

The mixed fracture micromechanism was noted in tested specimens of versions 3 (Fig. 2f) and 4 (Fig. 2h). The fracture surfaces comprise brittle cleavage areas of ceramic matrix neighbouring to ductile fracture ones of reduced nickel (Fig. 2f). Like for the NiO ceramics treated [4], this corresponds to higher cohesive strength between the particles of zirconia and nickel phase as compared to the singly reduced material.

#### The Treatment Temperature of 800 °C

In order to reduce the redox treatment duration, the behaviour of YSZ–NiO ceramics was estimated under redox cycling at 800 °C. During single exposition of the material in the Ar–5 vol% H_2_ mixture at this temperature (version 5 in Table 1), NiO particles are reduced completely within 1 h (Fig. 4а) by diffusion mechanism of much higher intensity as compared to version 1. Reduced Ni particles are dotted with nanopores. The NiO to Ni transformation is carried out rapidly with formation of comparatively coarse substructure (parameter *D* of reduced particles was measured as 64 nm, see Fig. 3(a)).

The X-ray data display the increase of the 2*Θ* angle for the material singly reduced at 800 °C. Consequently, the spacing between the planes in the atomic lattice (*d*) of the zirconia phase decreases, resulting in considerable growth of residual stresses (*σ*_r_) in the material of version 5 as compared to the as-received material (Fig. 3(b)).

The singly reduced material has somewhat lower strength as compared to as-received ceramics (Fig. 3(c), version 5) but substantially higher electrical conductivity as compared to the material treated at 600 °C (Fig. 3(d), versions 5 and 1, respectively). The mixed fracture micromechanism is recognized in the specimens tested (Fig. 4b). It is similar to the one after the redox treatment of YSZ–NiO ceramics at 600 °C. It also evidences that single reduction at 800 °C does not violate the integrity of the zirconia skeleton and a partial decrease in strength is caused by nickel phase transformation.

**Fig. 5:**
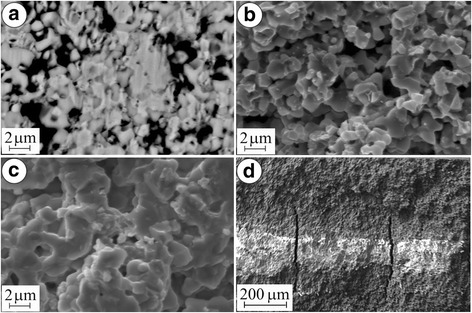
SEM microstructure (**a**) and microfractographs (**b**–**d**) for the material in version 6 (see Table 1)

Contrary to the positive effect of redox treatment of YSZ–NiO ceramics at 600 °C, a negative tendency for strength of YSZ–NiO ceramics during the treatment at 800 °C is noted (Fig. 3(c), version 6). In both cases of the treatment of YSZ–NiO ceramics, the resulting structures are similar (Fig. 5a as compared to Fig. 2e) except for the peculiar (of green colour) inner part of specimens after the treatment at 800 °C (Fig. 5d). As stated above, a kinetic mechanism of oxidation at this temperature intensifies the growth of unreduced volume of the anode during redox cycling.

Two different areas on the fracture surface picture of a specimen treated at 800 °C are observed. In the reduced layer, mixed fracture micromechanism comprising transgranular cleavage of zirconia particles and intergranular fracture along the boundaries of contacting nickel phase particles dominates (Fig. 5b). The nickel particles of smaller sizes had possibly agglomerated on zirconia particles. Being intact, they are separated by a network of the pores formed during the redox treatment. Thus, this fracture micromechanism is as energy expensive as the one revealed for the material after the redox treatment at 600 °C (Fig. 2f).

In the unreduced inner layer of the anode transgranular cleavage, a fracture of coarse zirconia and nickel oxide agglomerates dominates, and occasionally, the signs of intergranular fracture along the boundaries of particles of smaller size are observed (Fig. 5c).

In spite of similar levels of residual stresses in the zirconia phase that were estimated for the entire reduced volume (Fig. 3(b), version 5) and outer reduced layer (version 6), respectively, different structural factors affect the integrity of these materials.

The total strength of the material after redox treatment at 800 °C is decreased considerably with the array of microcracks in the bulk of the specimen (Fig. 5d) that have formed during the treatment normally to its surface. These microcracks are nucleated following a stress gradient on the boundaries between reduced and unreduced layers as a result of thermal expansion (thermal expansion coefficients for YSZ, NiO and Ni phases are 10.9 · 10^−6^, 14.1 · 10^−6^ and 16.4 · 10^−6^/K, respectively [14]). The reduced layer showed the value of specific electrical conductivity 3.4 · 10^5^ S/m (Fig. 3(d), version 6) which is satisfactory for SOFC anodes. However, this value is lower than that of the material singly reduced at 800 °C (version 5). Beside, the unreduced layer having very low electrical conductivity becomes a substantial obstacle in achieving the required electrochemical performance of the fuel cell.

Thus, contrary to the positive effect of redox treatment at 600 °C on strength and electrical conductivity of YSZ–NiO ceramics, such treatment at 800 °C causes the formation of anode structure with reduced outer and unreduced inner layers as well as the array of microcracks in the bulk of an anode initiated normally to its surface, what causes the loss of its integrity. The unreduced inner layer of the anode has unsatisfactory electrical conductivity.

## Conclusions

A cyclic treatment technique (redox cycling) comprising stages of material exposition in reducing and oxidizing high-temperature gas environments and intermediate degassing between these stages has been developed to improve the strength and electrical conductivity of YSZ–NiO ceramic anode substrates for solid oxide fuel cells. Based on the experimental data, we suppose that redox cycling at 600 °C is the most ideal for this material. The parameters of the treatment (reduction dwell time not less than 4 h under the pressure of the Ar–5 vol% H_2_ mixture of 0.15 MPa; oxidation dwell time 4 h) allow a structure to be formed which provides improved physical and mechanical properties of the material. In such a structure, according to the X-ray analysis, a substantial drop of residual stresses is achieved as compared to the one-time reduced material.
